# Sexual congruency in the connectome and translatome of VTA dopamine neurons

**DOI:** 10.1038/s41598-017-11478-5

**Published:** 2017-09-11

**Authors:** Amanda S. Chung, Samara M. Miller, Yanjun Sun, Xiangmin Xu, Larry S. Zweifel

**Affiliations:** 1 Department of Pharmacology, University of Washington, Seattle, WA 98195 USA; 2 Department of Anatomy and Neurobiology, University of California, Irvine, CA 92697 USA; 3 Department of Psychiatry and Behavioral Sciences, University of Washington, Seattle, WA 98195 USA

## Abstract

The ventral tegmental area (VTA) dopamine system is important for reward, motivation, emotion, learning, and memory. Dysfunctions in the dopamine system are linked to multiple neurological and neuropsychiatric disorders, many of which present with sex differences. Little is known about the extent of heterogeneity in the basic organization of VTA dopamine neurons with regard to sex. Here, we characterized the cell-specific connectivity of VTA dopamine neurons, their mRNA translational profile, and basic electrophysiological characteristics in a common strain of mice. We found no major differences in these metrics, except for differential expression of a Y-chromosome associated mRNA transcript, *Eif2s3y*, and the X-linked, X-inactivation transcript *Xist*. Of note, *Xist* transcript was significantly enriched in dopamine neurons, suggesting tight regulation of X-linked gene expression to ensure sexual congruency. These data indicate that the features that make dopamine neurons unique are highly concordant and not a principal source of sexual dimorphism.

## Introduction

Dopamine is a principal catecholamine neurotransmitter in the central nervous system, with dopamine producing neurons located in numerous brain regions including the ventral midbrain, hypothalamus, olfactory bulb, and retina^1^. Of these, dopamine producing neurons of the ventral midbrain located in the VTA have been among the most widely studied for their roles in reward, motivation, learning, and memory^2, 3^.

Alterations in the function of the midbrain dopamine system are broadly implicated in a diverse array of neurological and neuropsychiatric disorders, such as addiction, schizophrenia, obsessive compulsive disorder, attention-deficit hyperactivity disorder, depression, autism, and Parkinson’s disease^4, 5^. Many of these disorders have differential prevalence in males and females^6–8^ and sexual dimorphism in the dopamine neurotransmitter system has been well documented^9^.

Sexual dimorphism in the brain can arise from several principle sources, including gonadal sex steroids, and X- and Y-linked gene expression that impact the early wiring of neural circuits and modulate neural circuit function post-development. Sexually dimorphic sensory systems, such as the vomeronasal organ and dimorphic circuit nodes that impinge on non-dimorphic structures, are also a major source of variance between sexes^10^.

Activity patterns of dopamine neurons have been shown to be modulated by sex steroids^11, 12^ and the dopamine transporter that regulates neurotransmitter reuptake has also been shown to be modulated by sex steroids^11^. In addition, dopamine neurons receive direct synaptic input from brain regions previously demonstrated to be sexually dimorphic in their organization, including the medial preoptic area (MPA), bed nucleus of the stria terminalis (BNST), and the medial amygdala^13^. What remains to be determined is the extent to which the VTA dopamine system is intrinsically sexually dimorphic.

To address this question, we mapped inputs to dopamine neurons in a cell type-specific manner using a rabies viral tracing method^14^ in male and female mice. We analysed downstream connectivity of VTA dopamine neurons by monitoring Fos induction in downstream target regions following cell-specific activation of dopamine neurons using the stimulatory DREADD receptor, HM3Dq^15^. In addition, we performed mRNA profiling of actively translating mRNA selectively in dopamine producing neurons of male and female mice using the RiboTag strategy^16^. Finally, we analysed the intrinsic electrophysiological properties of genetically defined dopamine neurons using acute brain slices from male and female mice. Collectively, our data show that the basic features of the VTA dopamine system are highly correlated between sexes, and are not a major source of sexual dimorphism. These data suggest that the intrinsic properties of the VTA dopamine system in male and female mice is not a contributing factor to sexual mosaicism of the brain, and support the larger premise recently demonstrated in the human nervous system that there is no explicitly male or female brain^17^ and meta-analysis of data from male and female rats demonstrating a lack of differential variability^18^.

## Results

### Dopamine neuron connectivity

**Figure 1:**
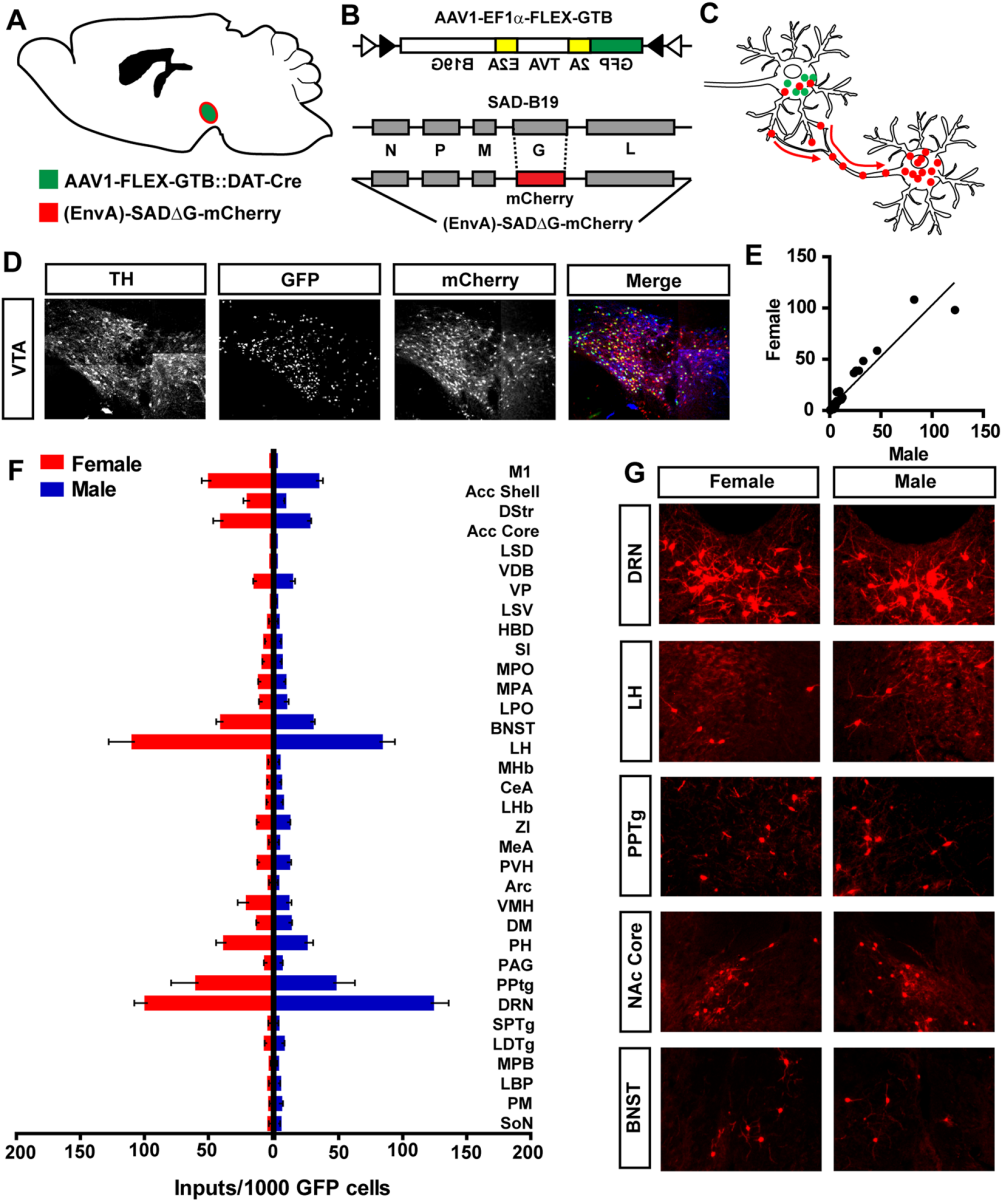
Cell-specific rabies viral tracing of dopamine neurons in male and female mice. (**A** and **B**) Male and female *Slc6a3* ^*Cre*^ mice were injected with AAV1-FLEX-GTB (green) two weeks prior to injection with EnvA-SAD-ΔG-mCherry (red). (**C**) Schematic illustrating cell-specific expression of GTB that allows for retrograde rabies viral expression of mCherry (atlas image from Paxinos and Franklin, 2001^46^). (**D**) Immunohistochemistry demonstrating expression of the dopaminergic marker tyrosine hydroxylase (TH), GFP, and mCherry in the VTA. (**E**–**G**) Numerous brain regions were identified as sending projections to the VTA that were highly correlated between male and female mice.

To examine the inputs to VTA dopamine neurons, we performed conditional rabies viral tracing^14^. To achieve cell-specificity, a Cre-dependent adeno-associated viral vector (AAV1-EF1α-FLEX-GTB) containing the tumor virus A (TVA) and the rabies glycoprotein (RG) was injected into the VTA of mice expressing Cre recombinase from the endogenous dopamine transporter locus (*Slc6a3* ^*Cre*/+^)^19^. Two weeks following AAV injection, mice were injected with the glycoprotein gene deleted virus containing the avian sarcoma leucosis virus glycoprotein EnvA (EnvA-SAD-ΔG-mCherry; Fig. 1A–C). After nine days, pseudotyped rabies virus injection inputs to VTA dopamine neurons were assessed by immunohistochemical analysis of mCherry expression. We observed no significant difference in the expression of GFP or mCherry in the VTA of male (n = 5) and female (n = 7) of *Slc6a3* ^*Cre*/+^ mice (Fig. 1D and Supplementary Figure S1A and B). Analysis of inputs to VTA dopamine neurons identified numerous brain regions, similar to those previously described^20^, and revealed an equivalent number of projections in female and male mice (962.4 ± 89.2, female vs. 802.2 ± 86.9, male; *P* = 0.24). Inputs to VTA dopamine neurons from specific brain regions were significantly correlated between sexes (Fig. 1E; *Slope* = 1.0 ± 0.06, *Pearson’s r* = 0.96, *P* < *0.0001*); no interaction between brain region and sex was observed (Fig. 1F and G; two-way ANOVA, *P* = 0.55).

**Figure 2:**
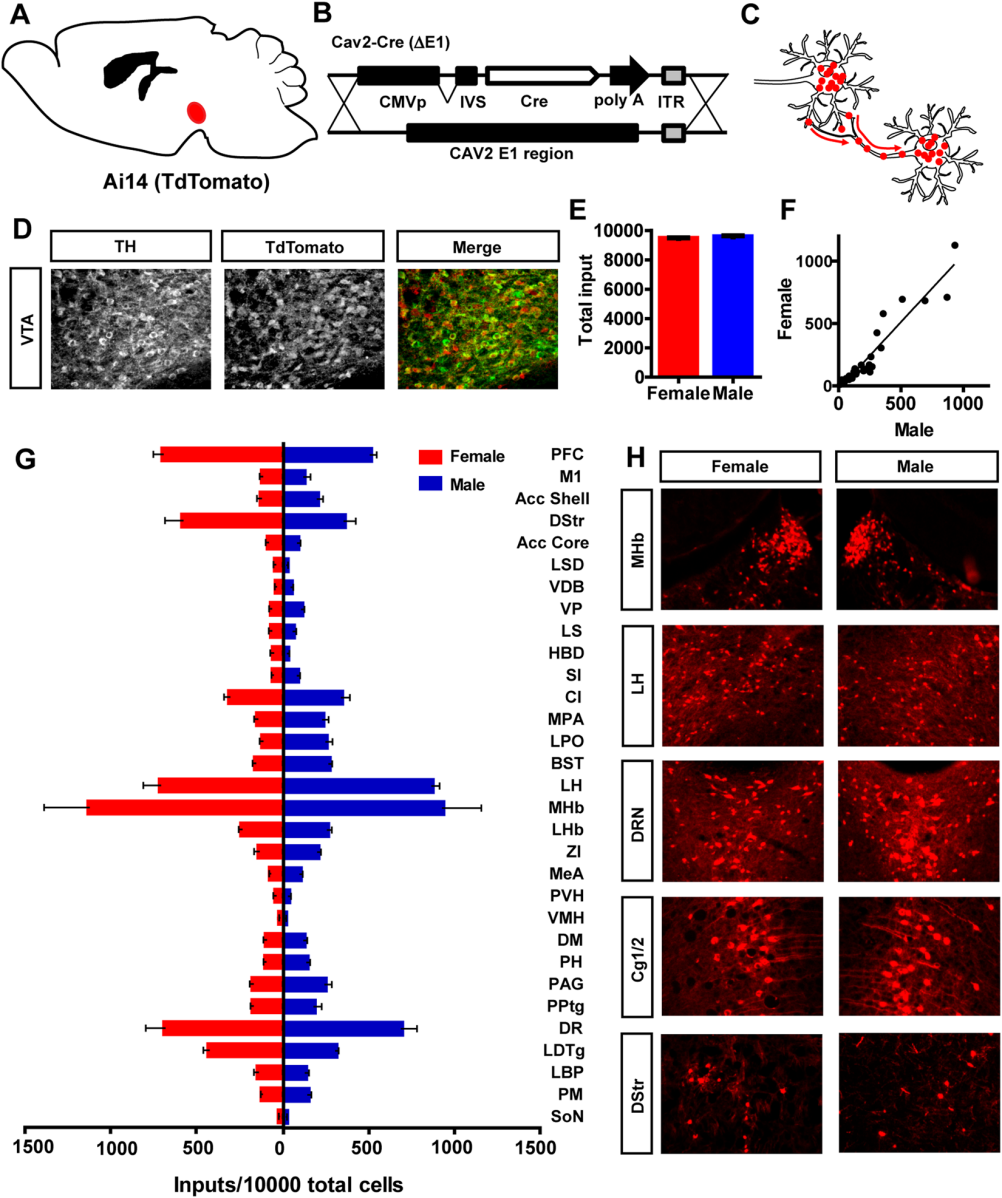
Non-cell specific CAV2-Cre viral tracing of dopamine neurons in male and female mice. (**A** and **B**) Male and female Ai14 reporter mice were injected with CAV2-Cre into the VTA. (**C**) Schematic retrograde transduction and tdTomato expression in inputs to the VTA. (**D**) Immunohistochemistry demonstrating expression of the dopaminergic marker TH and tdTomato in the VTA. (**E**–**G**) Numerous brain regions were identified as sending projections to the VTA that were highly correlated between male and female mice.

In addition to cell-specific mapping using the rabies viral approach, we also mapped inputs to the VTA using a non-cell specific approach. To achieve this, we utilized the retrograde transducing virus canine adenovirus (CAV2) containing an expression cassette for Cre recombinase (CAV2-Cre)^21^. CAV2-Cre was injected into the midbrain of male (n = 5) and female (n = 5) Ai14 reporter mice (*Gt(ROSA)26Sor* ^*tm(CAG-tdTomato)Hze*^-^22^; Fig. 2A–D), Cre-mediated expression of tdTomato was quantified across multiple brain regions, similar to our quantification of rabies virus labelling (Fig. 2E–H). Although CAV2-Cre labelled a larger number of cells, the results were similar with regard to sex. Region-specific inputs were significantly correlated between sexes (Figure S1H; *Slope* = 1.08 ± 0.08, *Pearson’s r* = 0.95, *P* < *0.0001*); no interaction between brain region and sex was observed (Fig. 2G; two-way ANOVA, *P* = 0.22).

**Figure 3:**
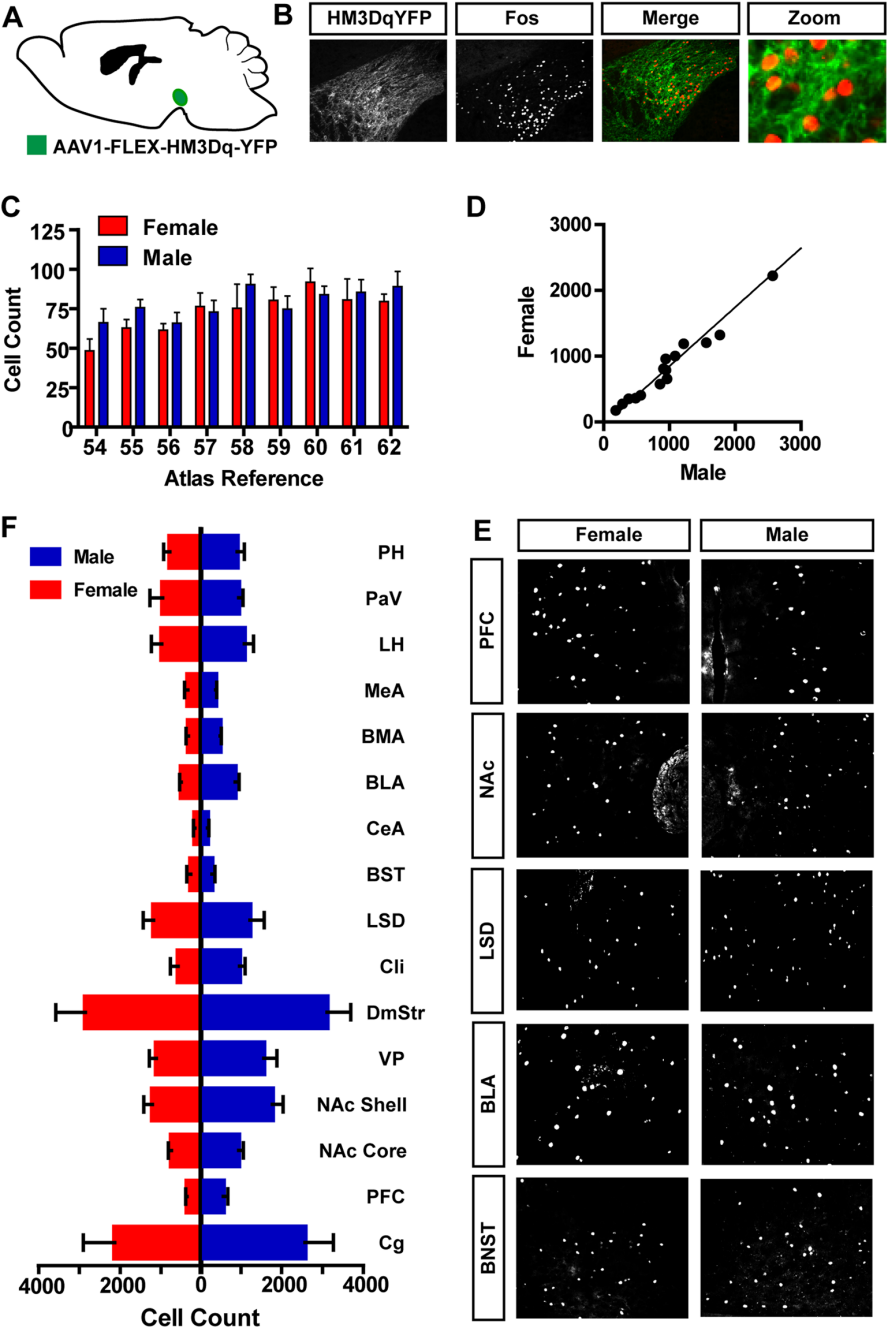
Activation of dopamine neurons induces cFos across multiple brain regions in male and female mice. (**A**) Schematic demonstrating the injection of AAV1-FLEX-HM4Dq-YFP into the VTA of female and male *Slc6a3* ^*Cre*^ mice. (**B**) Immunohistochemistry demonstrating cFos expression in VTA dopamine neurons following injection of CNO. (**C**) Rostral-caudal distribution of cFos staining across the VTA following CNO injection is not different between males and females. (**D**–**F**) cFos expression across multiple brain regions following CNO injection is highly correlated between female and male mice.

To establish the extent to which the basic connectivity of VTA dopamine neuron projections are similar, or dissimilar between male and female mice, we activated dopamine producing neurons using the stimulatory DREADD receptor HM3Dq^15^ and assayed for induction of the immediate early gene *Fos* in downstream target structures. Two-weeks following injection of AAV1-FLEX-HM3Dq-YFP into the VTA of *Slc6a3* ^*Cre*/+^ female (n = 4) and male (n = 5) mice (Fig. 3A) we injected mice with the DREADD receptor agonist clozapine-N-oxide (CNO, 1 mg/kg i.p.) to induce neuronal activation. CNO induced robust Fos expression in the VTA along the rostral-caudal axis that did not differ between male and female mice (Fig. 3B and C; two-way ANOVA, *P* = 0.75). Similarly, we observed significantly correlated Fos expression across multiple downstream brain regions between sexes (Fig. 3D; *Slope* = 0.9 ± 0.04, *Pearson’s r* = 0.99, *P* < *0.0001*); no interaction between sex and brain region was observed (Fig. 3E and F; two-way ANOVA, *P* = 0.99). Projections to the Fos-positive areas were confirmed through expression of the synaptic marker synaptophysin-GFP in VTA dopamine neurons by injecting Cre-dependent virus (AAV1-FLEX-Synapto-GFP)^23^ into *Slc6a3* ^*Cre*/+^ mice (Figure S2A and B).

### Translational profile of VTA dopamine neurons

**Figure 4:**
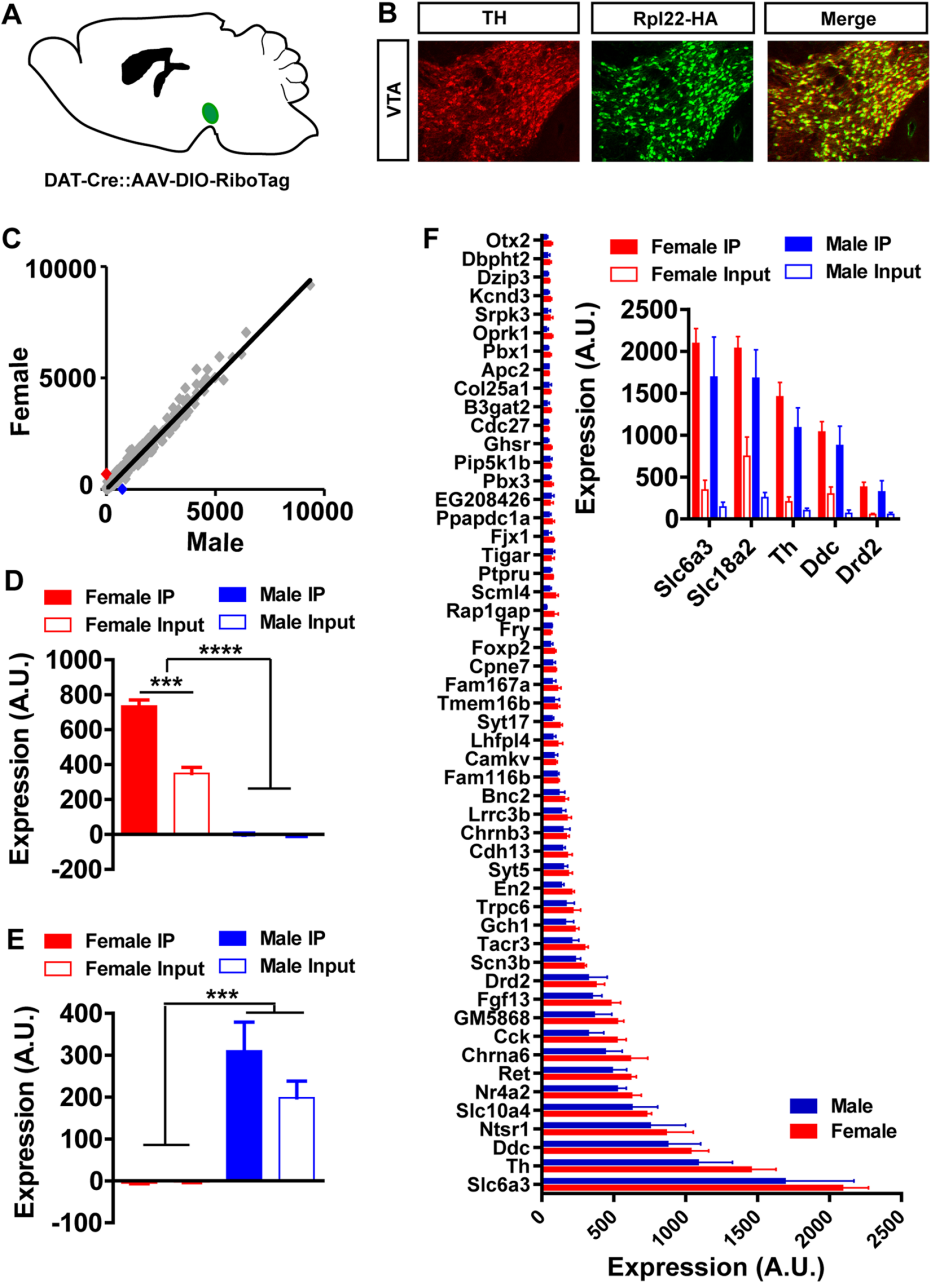
Translatome analysis of dopamine neurons in female and male mice. (**A**) Schematic demonstrating the injection of AAV1-FLEX-Rpl22-HA into the VTA of female and male *Slc6a3* ^*Cre*^ mice. (**B**) mRNA levels are highly correlated between females and males. (**C**,**D**) Two genes, *Xist* (red, C) and *Eif2s3y* (blue, D) are highly differentially expressed in male and female mice. *Xist* is significantly enriched in dopamine neurons of female mice (**C**). (**F**) mRNA with a greater than 4-fold enrichment in dopamine neurons, including those necessary for dopamine synthesis and release (inset), are not different between females and males.

To determine the extent to which actively translating mRNA in dopamine neurons is similar between male and female mice, we utilized a RiboTag strategy that allows for cell-specific immuno-isolation of polyribosomal mRNA^16^. *Slc6a3* ^*Cre*/+^ mice were injected into the VTA with an AAV vector containing a Cre-dependent expression cassette for the affinity tagged ribosomal protein Rpl22 (AAV1-EF1α-Rpl22-HA^24^; Fig. 4A). Three weeks following viral injection to allow for Rpl22-HA expression (Fig. 4B), the midbrain was microdissected and polyribosomes were immunoprecipitated (IP). Polyribosome-associated mRNA was reverse transcribed and cDNAs from male (n = 4) and female (n = 3) mice were analysed by Illumina microarray. Analysis of all IP transcripts from male and female mice revealed significantly correlated translatomes (Fig. 4C; *Slope* = 0.98 ± 0.05, *Pearson’s r* = 0.96, *P* < *0.0001*). Of the > 18,000 probes, only two, the X-linked *Xist* and the Y-chromosome gene *Eif2s3y*, were identified as significantly different between female and male translatomes (Fig. 4D and E). Of these, only *Xist* was significantly enriched in dopamine neurons (Fig. 4D). Analysis of enrichment (IP/input) showed 52 genes with greater than 4-fold enrichment, including canonical markers for dopamine synthesis and release (Fig. 4F). There was no significant interaction between gene and sex of enriched markers (two-way ANOVA, *P* = 0.99).

### Intrinsic electrophysiological properties of dopamine neurons

**Figure 5:**
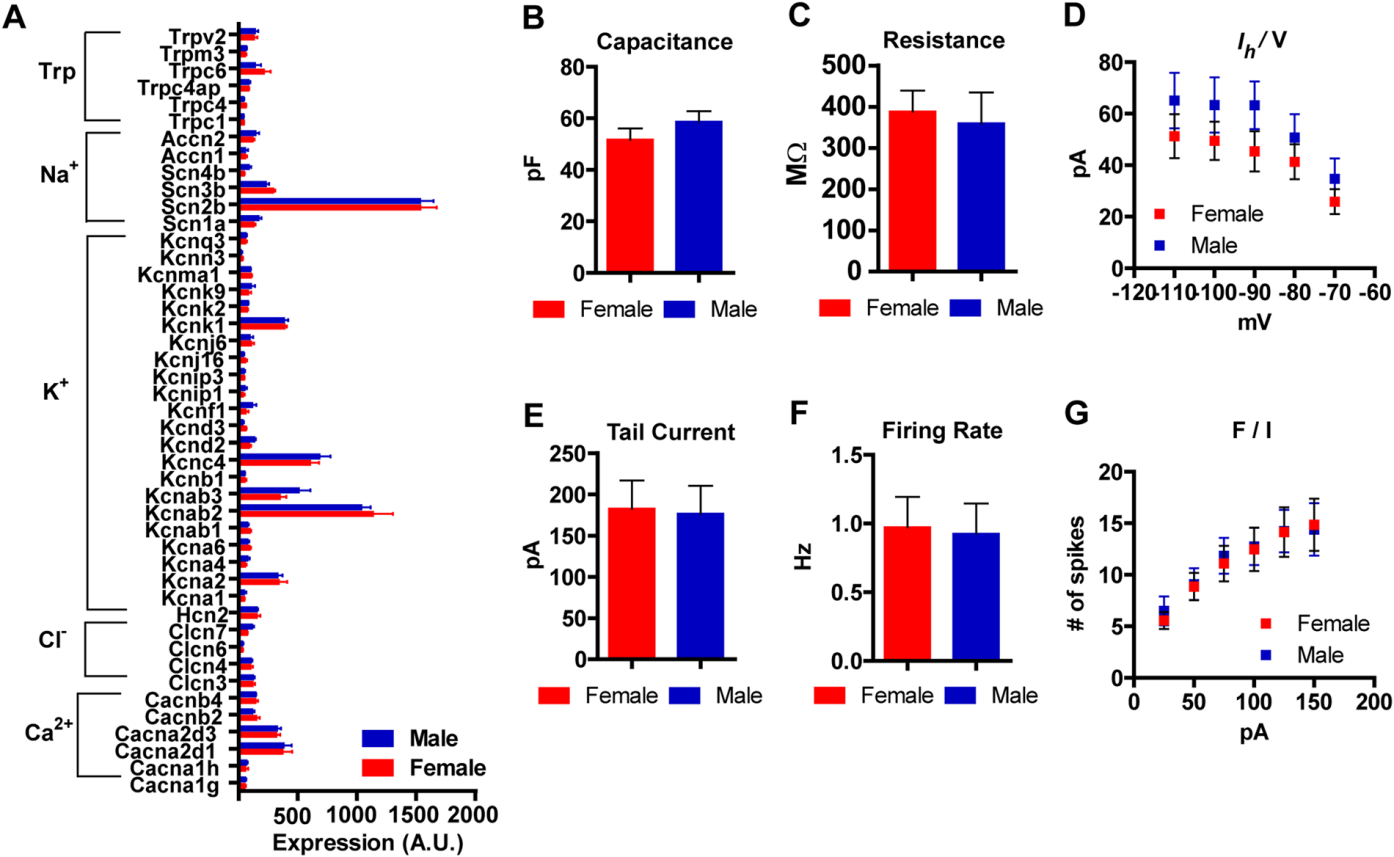
Basic electrophysiological profiles and mRNA expression for ion channels in male and female mice. (**A**) mRNA for non-ligand gated ion channels. (**B** and **C**) Capacitance and input resistance in genetically identified dopamine neurons. (**D**) Input-output curve for *I*_*h*_ current. (**E**) Tail current is not different between female and male mice. (**F**) Firing rate in response to increasing current injection F/I.

Our connectivity and translatome analysis are consistent with equivalent organization of the VTA dopamine system in male and female mice. To explore this further, we analysed the translational profile of genes encoding ion channels that are known to regulate dopamine neuron activity and intrinsic excitability, as well as genes encoding neurotransmitter and neuropeptide receptors. Numerous ion channel subunits were expressed in dopamine producing neurons, but none were found to be differentially expressed in female and male mice (Fig. 5A). To establish which of these genes have specific expression in dopamine neurons we pooled male and female samples; twelve genes showed significant enrichment (Supplementary Figure S3A; Wilcoxon signed rank test, theoretical median 1; p < 0.05). Of these, six showed 4-fold or greater enrichment (*Clcn6*, *Kcnd3*, *Kcnn3*, *Scn3b*, *Trpc4*, and *Trpc6*).

To confirm these observations, we recorded the intrinsic electrophysiological properties of midbrain dopamine neurons using whole-cell patch clamp recordings from acute brain slices. Adult female and male *Slc6a3* ^*Cre*/+^;*Gt(ROSA)26Sor* ^*tm(CAG-tdTomato)Hze*^ mice (8 weeks of age) were utilized to genetically identify dopamine producing neurons by tdTomato fluorescence. Basic features including capacitance and input resistance did not differ between females and males (Fig. 5B and C). Afterhyperpolarization induced current (*I*_*h*_), a prominent feature of many, but not all dopamine neurons^25^, also did not differ (Fig. 5D; Supplementary Figure S4A), consistent with equivalent expression of the hyperpolarization activated, cyclic nucleotide-gated potassium channel HCN2 (*Hcn2*, Fig. 5A). We also observed equivalent expression of the small conductance calcium-activated potassium channel, SK3 (*Kcnn3*, Fig. 5A), known to be enriched in dopamine neurons^26, 27^. We did not observe differences between sexes in tail currents (Fig. 5E; Supplementary Figure S4B), known to be principally mediated by SK3 channels^26^. Intrinsic pacemaker firing (Fig. 5F; Supplementary Figure S4C) and current-induced spike firing (Fig. 5G; Supplementary Figure S4D) also did not differ between sexes.

**Figure 6:**
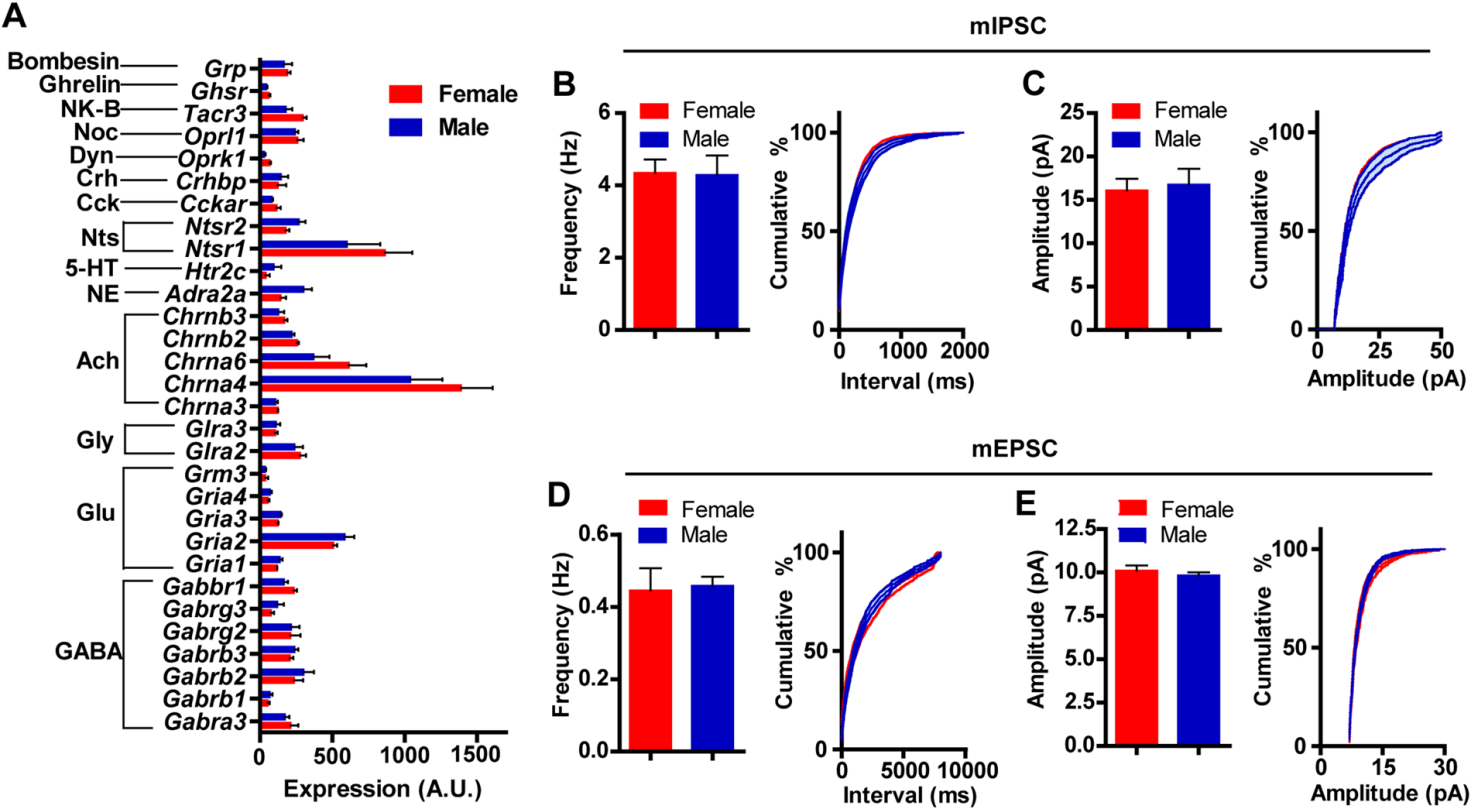
Basic electrophysiological profiles and mRNA expression for neurotransmitter receptors in male and female mice. (**A**) Neurotransmitter and neuropeptide mRNA levels in dopamine neurons. (**B** and **C**) mIPSC frequency (**B**) and amplitude (**C**). (**D**,**E**) mEPSC frequency (**D**) and amplitude (**E**).

Similar to ion channel subunits, analysis of neurotransmitter and neuropeptide receptors did not reveal sex-specific differences in actively translating mRNA (Fig. 6A). Twelve genes were found to be enriched (>2-fold); two neurotransmitter receptor subunits (*Chrna6* and *Chrnb3*) and four neuropeptide receptors (*Oprk1*, *Ghsr*, *Ntsr1*, and *Tacr3*) were highly enriched (Supplementary Figure S3B). Consistent with our input mapping, analysis of excitatory and inhibitory synaptic connectivity, as measured by miniature inhibitory and excitatory postsynaptic currents (mIPSC and mEPSC), showed equivalent frequency and amplitude of mIPSCs (Fig. 6B and C; Supplementary Figure S4E) and mEPSCs (Fig. 6D and E; Supplementary Figure S4F) in male and female dopamine neurons.

## Discussion

In this study we have analysed the cell-type specific connectivity of the VTA midbrain dopamine system, the translational profile of these neurons, and their basic electrophysiological properties in both male and female mice. Our study was specifically designed to not account for potential effects of circulating sex steroids, or sexual dimorphism in brain regions projecting to the VTA in order to establish whether analysis of this system in an unbiased manner would reveal differences in variability that may subsequently be accounted for by sex steroid differences or dimorphic organization of inputs to the regions. Our analysis reveals a highly congruent organization with no measurable differences between sexes relating to within subjects variability.

Our cell-specific input analysis using Cre-dependent viral rabies tracing is consistent with numerous previous reports; however, previous studies did not investigate the extent of sex differences^20, 28–31^. Our findings match previous observations that the highest density of inputs to dopamine neurons arise from the DRN, BNST, NAc, PPTg, and LH. Further, our output analysis using conditional expression of the stimulatory DREADD receptor HM4Dq in dopamine neurons confirmed previous observations of Fos expression following chemogenetic activation of dopamine neurons^32^. Interestingly, although we did not observe sex-specific differences in the input and output relationships of VTA dopamine neurons we did identify numerous brain regions projecting to the VTA and receiving input from the VTA that have been previously described as being sexually dimorphic in their organization. These include the BNST, medial amygdala, prefrontal cortex, and hypothalamus^13^. These findings are consistent with an organization in which sexually dimorphic nodes intersect with the non-dimorphic VTA dopamine system that are likely to give rise to sex-specific motivated behaviours, such as those described recently for MPA regulation of motivated behaviours in female mice^33^.

Similar to our analysis of the connectome of dopamine neurons, examination of the translatome of these neurons using the RiboTag method revealed enrichment of actively translating mRNAs that are consistent with previous analysis of gene expression in dopamine producing neurons^34–38^. We found no sex-specific differences in genes that were preferentially expressed in dopamine neurons; however, our analysis did reveal two sexually dimorphic genes: *Xist* and *Eif2s3y*. *Xist* is an X-linked gene whose purpose is to inactivate one of the two X chromosomes in females, to prevent gene dosage effects^39^. The reason for our observation of *Xist* transcript being associated with Rpl22 is unclear, but likely reflects the previous observations that *Xist* interacts with ribonuclear proteins involved in RNA splicing^40^ and the recently identified role of Rpl22 in RNA splicing^41^. We find *Xist* to be >2-fold enriched in VTA dopamine neurons, suggesting that this cell type potently regulates X-linked gene expression to ensure a lack of gene dosage effects, further supporting the notion that the dopamine neurons of the VTA are programmed to ensure highly concordant gene expression. It is not surprising that *Eif2s3y* is only expressed in males. We find the X-linked homolog of *Eif2s3y*, *Eif2s3x* is expressed in both male and female dopamine neurons and is not enriched within these cells. Although the broad expression of *Eif2s3y* and *Eif2s3x* in the brain has been reported, the exact function of these genes is largely unknown^42^.

In agreement with our findings that the translational profile of dopamine neurons is equivalent between male and female mice, we did not find sex-specific differences in transcripts for ligand and non-ligand gated ion channels, and neurotransmitter and neuropeptide associated G-protein coupled receptors. Additionally, we did not find differences in the intrinsic electrophysiological properties of dopamine neurons. Although our findings indicate that the basic organization of the dopamine system is not different between male and female mice, it does not preclude the existence of differences within the system. It has been previously shown that psychostimulant drugs such as cocaine result in differential behavioural outcomes in male and female rats^43^ and mice^11^. These behavioural differences likely reflect differential regulation of dopamine release and reuptake by sex steroids^11, 44, 45^, or differences in the *in vivo* electrophysiological properties of dopamine neurons in response to sex hormones^11, 12^. Hormonal regulation of dopamine release in the PFC of male rats has also demonstrated^46^.

In summary, our data support the conclusion that the organizational principles of the VTA dopamine neurons are not sexually dimorphic, but rather likely reflect a fundamentally basic function of this system for reward processing, motivation, and emotional regulation. Based on the framework that sexual dimorphism can arise from several principal sources, including X- and Y-linked gene expression, sex steroids, sexually dimorphic inputs, and developmental programming, we conclude that the major sources that contribute to the previously ascribed dimorphism of the VTA dopamine system arise from sources outside the developmental organization of VTA dopamine neurons. These sources include hormonal regulation and circuits nodes upstream of dopamine neurons. Our data support the conclusion that the organization of the VTA dopamine system is invariant with regard to sex role allowing it to function as a basic facilitator of reward and motivational processes. Such an organization allows for flexibility in the control of sex-specific behaviours without the need for evolving multiple, intendent motivational systems.

## Methods

### Animals

All methods and experiments were approved by the University of Washington Institutional Animal Care and Use Committee. All experiments were performed in accordance to guidelines and regulations. *Gt*(*ROSA*)*26Sor* ^*tm*(*CAG-tdTomato*)*Hze*^ mice aged 10 weeks were used for all CAV2-Cre experiments. *Slc6a3* ^*Cre*/+^mice aged 10 weeks were used for all rabies virus, HM3Dq, and RiboTag experiments. *Slc6a3* ^*Cre*/+^;*Gt*(*ROSA*)*26Sor* ^*tm*(*CAG-tdTomato*)*Hze*^ mice aged 8 weeks were used for electrophysiology experiments. Both male and female mice were used in these experiments.

### Viral Injection

#### Connectivity

CAV2-Cre: All mice were anesthetized using isoflurane and stereotaxically injected bilaterally with CAV2-Cre (0.5 uL/side) into the VTA. Stereotaxic injection coordinates from bregma in mm, A-P: −3.25*x, M-L: ± 0.5, D-V: −4.5 (x = lambda:bregma distance/4.21) for the VTA. Mice were allowed to recover for two weeks before perfusion.

#### Rabies

All mice were anesthetized using isoflurane and stereotaxically injected bilaterally with AAV1-EF1α-FLEX-GTB (0.5 uL/side) into the VTA. After two weeks, mice were injected bilaterally with EnvA-SAD-ΔG-mCherry (0.5 uL/side) into the VTA. Stereotaxic injection coordinates from bregma in mm, A-P: −3.25*x, M-L: ± 0.5, D-V: −4.5 (x = lambda:bregma distance/4.21) for the VTA. Mice were allowed to recover for 9 days before perfusion.

#### HM3Dq

All mice were anesthetized using isoflurane and stereotaxically injected bilaterally with AAV1-HM3Dq-YFP (0.5 uL/side) into the VTA. Stereotaxic injection coordinates from bregma in mm, A-P: −3.25*x, M-L: ± 0.5, D-V: −4.5 (x = lambda:bregma distance/4.21) for the VTA. Mice were allowed to recover for 2 weeks. Mice were then habituated to syringe injection with saline injections daily for 3 days. On the fourth day, mice were injected with 1 mg/kg of CNO and perfused 2 hours later.

#### RiboTag

All mice were anesthetized using isoflurane and stereotaxically injected bilaterally with AAV1-DIO-Rpl22-HA (0.5 uL/side) into the VTA. Stereotaxic injection coordinates from bregma in mm, A-P: −3.25*x, M-L: ± 0.5, D-V: −4.5 (x = lambda:bregma distance/4.21). Mice were allowed to recover for four weeks before tissue collection.

### Histology

Mice were anesthetized with 50 mg/kg of Beuthenasia and perfused with phosphate-buffered saline (PBS) and 4% paraformaldehyde. Whole brains were dissected and fixed overnight in paraformaldehyde, followed by immersion in a 30% sucrose solution for at least 48 hours. Brains were frozen in OCT at −20 degrees Celsius and sectioned coronally on a cryostat in 30 um sections. Sections were then stored in PBS and 0.1% sodium azide until immunostaining and/or mounting onto slides for imaging.

### Immunostaining

cFos: Every other section from the entire brain was washed in 1x tris buffered solution (TBS) + 0.3% TritonX 100 (TBST) with 3% donkey serum for 30 minutes. Sections were then incubated overnight at 4 degrees Celsius or for 4 hours at room temperature in primary antibody (rabbit anti cfos, 1:2000, CalBiochem). This was followed by a 3x wash in TBS for 10 minutes and a 1 hour incubation at room temperature in secondary antibody conjugated to Cy3 or AF-488 at a 1:200 dilution. Finally, sections were washed in 1x TBS 3 more times before mounting onto slides.

RiboTag: Primary antibodies of mouse anti-HA (1:1000, ABM) and rabbit anti-TH (1:1000, Millipore) were used.

### Image Analysis

Fluorescent images of whole sections were acquired at 10x magnification (Keyence BZ-X710) and organized based on corresponding atlas reference figures (Mouse Brain Atlas, Franklin and Paxinos). Cells were then counted manually with the exception of cFos, which was counted using ImageJ software. This was done by analysing particles within individually drawn outlines of brain structures based on atlas reference figures.

### RiboTag

Brain tissue from the VTA area was collected using a tissue punch and homogenized, as previously described^16^. Tissue was then incubated with 5 ul of anti-HA primary antibody (Covance) for four hours at 4 degrees Celsius, followed by overnight incubation with 200 ul of magnetic beads (Pierce). Next, RNA-conjugated beads were washed using a high salt buffer and the RNA was extracted from the magnetic beads. RNA was then purified using a RNeasy Plus Micro kit (Qiagen).

To confirm efficient enrichment for dopaminergic markers, qRT-PCR analysis was performed for *Slc6a3*. mRNA in IP versus input was quantified using a Ribogreen RNA kit (Invitrogen) and converted to cDNA using Superscript IV and oligo dT primers (Invitrogen). TaqMan primers (Applied Biosystems) for *Slc6a3* were used to measure gene expression. Expression was quantified using the Ct values normalized to *Actb* (ΔCt). Fold enrichment of IP over input was calculated for using 2^−ΔΔCt^. Of the four male and four female mice injected and processed, one female did not shown enrichment for *Slc6a3* mRNA and was excluded from further analysis.

For samples intended for microarray analysis, RNA was amplified (Ovation PicoSL WTA RNA Amplification System), purified (Qiagen MinElute Reaction Cleanup Kit), and the quantity was measured again using a nanodrop. Confirmation of dopamine marker enrichment was again performed using TaqMan primers, after which biotinylation (Encore BiotinIL) and purification (Qiagen) was done. Samples were then checked for quality (Agilent RNA 6000 Nano) and hybridized to the microarray chip (Illumina Mouse 8 Channel V2). Microarray data was read and analysed using Illumina iScan (GenomeStudio). Data were exported to Excel (Microsoft) and correlational analysis was performed. Genes with a relative expression greater than 4-fold (IP/input) were designated as highly enriched and further analysed for sex-specific differences.

### Slice Electrophysiology

Whole-cell recordings were made using an Axopatch 700B amplifier (Molecular Devices) with filtering at 1 KHz using 4-6 MΩ electrodes filled with an internal solution containing (in mM): 130 K-gluconate, 10 HEPES, 5 NaCl, 1 EGTA, 5 Mg-ATP, 0.5 Na-GTP, pH 7.3, 280 mOsm. Horizontal brain slices (200 μm) were prepared from 8 week old mice in an ice slush solution containing (in mM): 92 NMDG, 2.5 KCl, 1.25 NaH_2_PO_4_, 30 NaHCO_3_, 20 HEPES, 25 glucose, 2 thiourea, 5 Na-ascorbate, 3 Na-pyruvate, 0.5 CaCl_2_, 10 MgSO_4_, pH 7.3–7.4. Slices recovered for ~12 min in the same solution at 32 degrees Celsius and then were transferred to a room temperature solution including (in mM): 92 NaCl, 2.5 KCl, 1.25 NaH_2_PO_4_, 30 NaHCO_3_, 20 HEPES, 25 glucose, 2 thiouria, 5 Na-ascorbate, 3 Na-pyruvate, 2 CaCl_2_, 2 MgSO_4_. Slices recovered for an additional 60 min. All solutions were continually bubbled with O_2_/CO_2_, and all recordings were made in ACSF at 32 degrees Celsius continually perfused over slices at a rate of ~2 ml/min. Ih currents were induced by 2-s hyperpolarizing voltage steps from −70 mV to −120 mV by 10 mV increments. SK currents were induced by depolarizing voltage steps from −70 to 0 mV. Capacitance measurements were calculated by software using 5 mV hyperpolarizing steps (Clampex).

For recording miniature excitatory postsynaptic currents, electrodes were filled with an internal solution containing (in mM): 130 K-gluconate, 10 HEPES, 5 NaCl, 1 EGTA, 5 Mg-ATP, 0.5 Na-GTP, pH 7.3, 280 mOsm, and 200 μM picrotoxin was bath applied through the ACSF to block inhibit GABAA receptor-mediated events. For recording spontaneous inhibitory postsynaptic currents, electrodes were filled with an internal solution containing (in mM): 135 KCl, 12 NaCl, 0.05 EGTA, 100 HEPES, 0.2 Mg-ATP, 0.02, Na-GTP (include pH and osmolarity here); 2 mM kynurenic acid was bath applied through the ACSF to block glutamatergic synaptic transmission. For all miniature current recordings, cells were clamped at a holding potential of −60 mV for a minimum of 5 minutes and were recorded in the presence of 1 mM tetrodoxin (TTX) to block action potentials. Access resistance was monitored throughout all experiments.

### Statistical Analysis

All statistical analysis was done using Prism (GraphPad).

### Data Availability

All data are available upon request.

## Electronic supplementary material

Supplementary Information
