# Supplementary material for: Sexual congruency in the connectome and translatome of VTA dopamine neurons
Source: Sci Rep. 2017 Sep 11;7:11120. doi: 10.1038/s41598-017-11478-5 (PMC5593921; doi:10.1038/s41598-017-11478-5)
Supplement: Supplementary file 1 — Supplementary Information [file 41598_2017_11478_MOESM1_ESM.pdf]

Supplementary information for: Sexual congruency in the connectome and translatome of VTA dopamine neurons

Authors: Amanda S. Chung, Samara M. Miller, Yanjun Sun, Xiangmin Xu, and Larry S. Zweifel

Supplementary Figure Legends

Supplementary Figure S1: Expression of GFP from AAV1-FLEX-GTB in the VTA and overlap with TH. A) Rostral to caudal expression of GFP in the VTA of male and female mice. No difference in the distribution was observed. B) Overlapping expression of TH and GFP (TH/GFP) in the VTA, GFP and TH (GFP/TH), TH and mCherry (TH/mCherry), and mCherry and TH (TH/mCherry) is not different in male and female mice.

Supplementary Figure S2: Viral mapping of VTA dopamine neuron projections. AAV1-FLEX-Synaptophysin-GFP was injected into the VTA *Slc6a3*$^{Cre/+}$ mice to confirm projections identified in Fos expression analysis. A) 10X stitched images of Synaptophysin-GFP in representative rostral to caudal brain regions next to brain atlas image (Paxinos and Franklin, 2001). B) Zoomed images from 10X stitched images showing Synaptophysin-GFP in target regions.

Supplementary Figure S3: Enriched expression of ion channels and neurotransmitter receptors in the VTA. A) Ion channels with enriched expression in the VTA (male and female mice pooled). Dashed red line indicates enrichment (IP/input) of one, indicating equivalent expression in dopamine and non-dopamine neurons. B) Enriched expression of neurotransmitter and neuropeptide receptors in the VTA.

Supplementary Figure S4: Representative traces from electrophysiology recordings from male and female mice. A) Representative I_h currents. B) Representative tail currents. C) Representative action potential firing and waveforms. D) Representative action potential firing following depolarizing current injections. E) Represnetative mIPSCs. F) Representative mEPSCs.

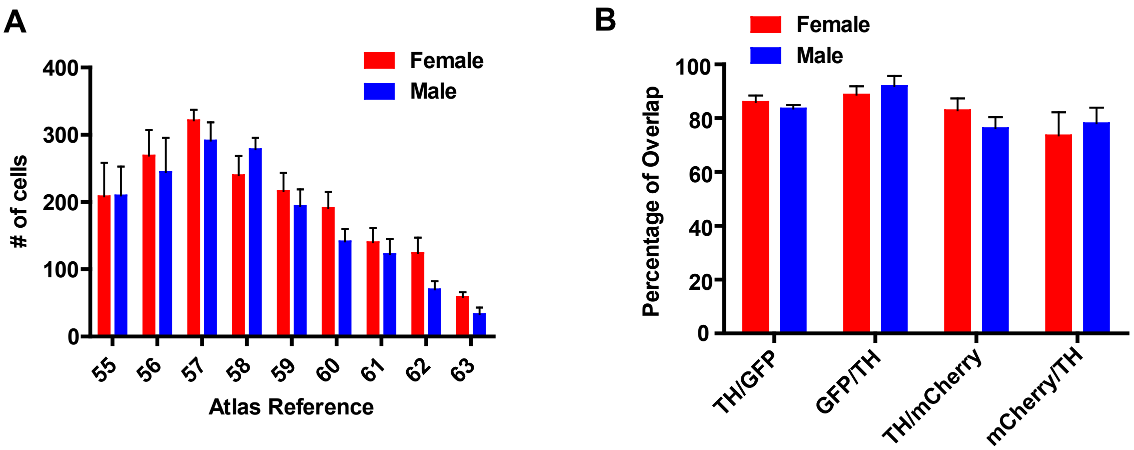
A
Female
Male
400
300
200
100
0
# of cells
55
56
57
58
59
60
61
62
63
Atlas Reference
B
Female
Male
100
80
60
40
20
0
Percentage of Overlap
TH/GFP
GFP/TH
TH/mCherry
mCherry/TH

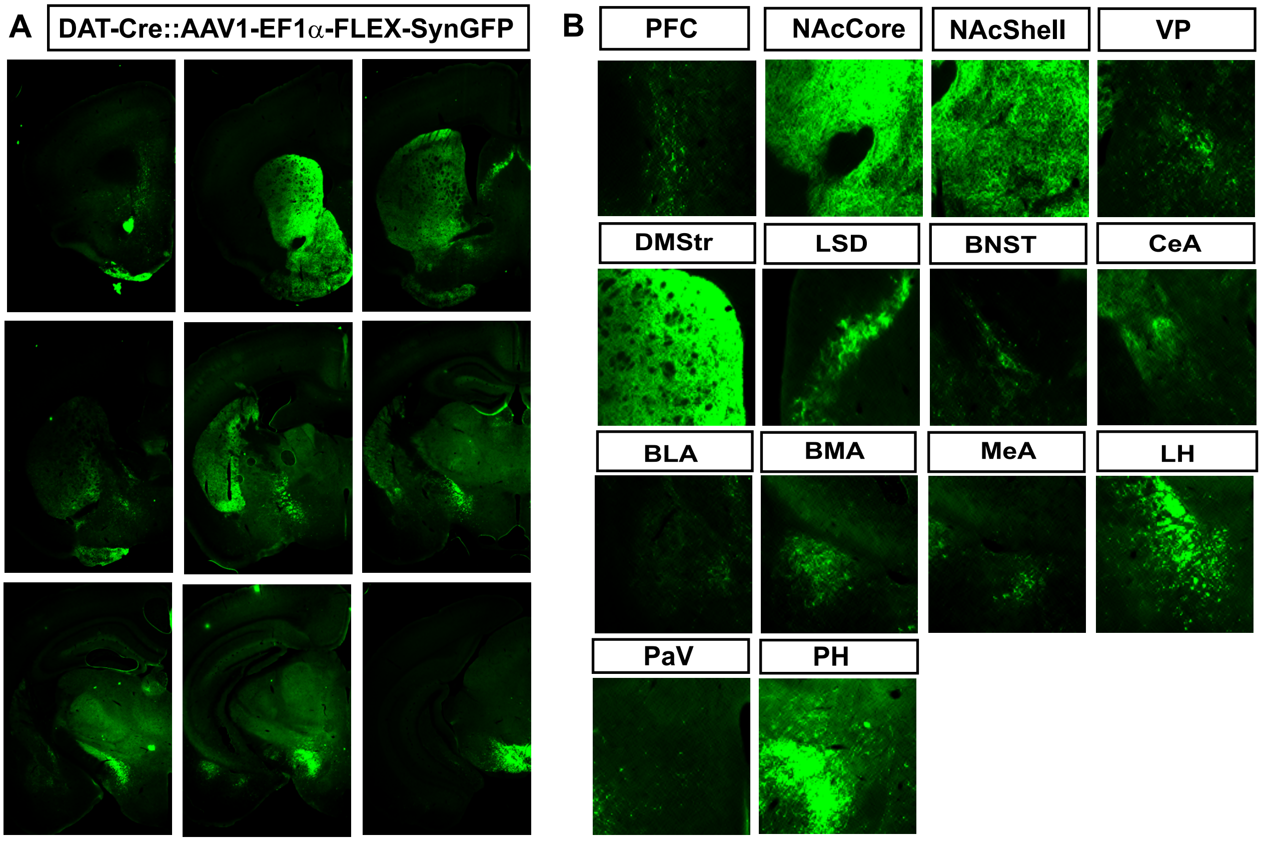
A
DAT-Cre::AAV1-EF1α-FLEX-SynGFP
B
PFC
NAcCore
NAcShell
VP
DMStr
LSD
BNST
CeA
BLA
BMA
MeA
LH
PaV
PH

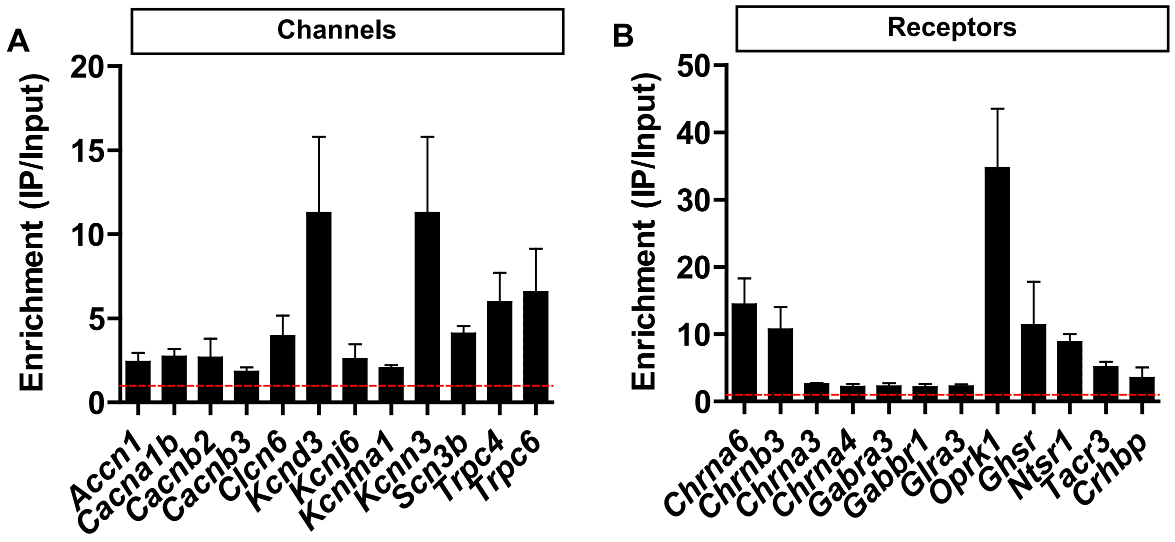

A
Channels
Enrichment (IP/Input)
20
15
10
5
0
Accn1
Cacna1b
Cacnb2
Cacnb3
Clcn6
Kcnd3
Kcnj6
Kcnma1
Kcnn3
Scn3b
Trpc4
Trpc6
B
Receptors
Enrichment (IP/Input)
50
40
30
20
10
0
Chrna6
Chrnb3
Chrna3
Chrna4
Gabra3
Gabbr1
Glra3
Oprk1
Ghsr
Ntsr1
Tacr3
Crhbp

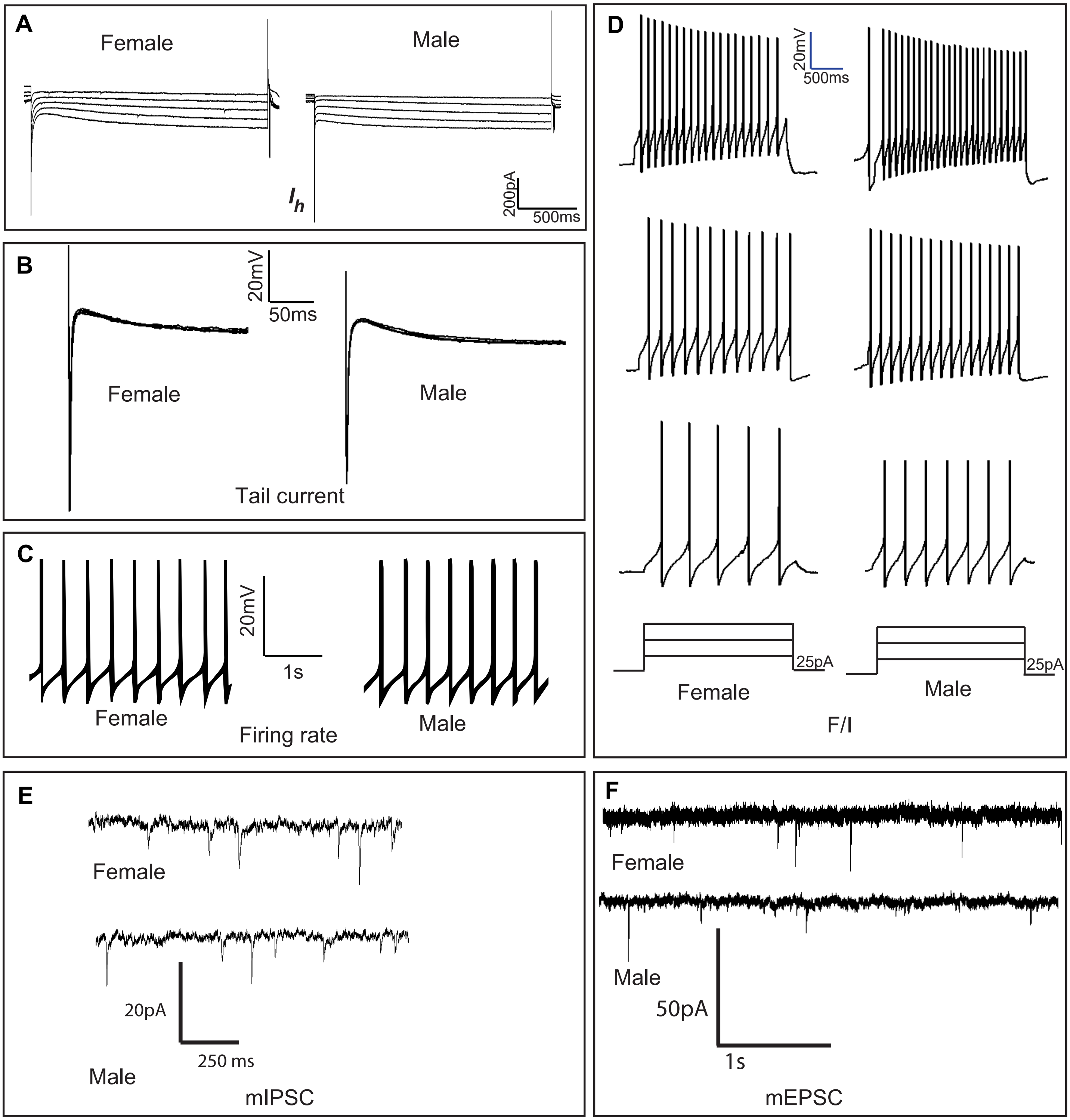

A
Female
Male
I_h
200pA
500ms
B
20mV
50ms
Female
Male
Tail current
C
20mV
1s
Female
Firing rate
Male
D
20mV
500ms
25pA
25pA
Female
Male
F/I
E
Female
20pA
250 ms
Male
mIPSC
F
Female
Male
50pA
1s
mEPSC
